# New method of sentinel lymph node biopsy in transoral robotic surgery for oropharyngeal squamous cell carcinoma

**DOI:** 10.6061/clinics/2018/e550s

**Published:** 2018-11-27

**Authors:** Marco Aurélio V Kulcsar, Natasha Sobreira Canovas, Vergilius Jose Furtado de Araujo-Neto, Jorge Du Ub Kim, Claudio Roberto Cernea

**Affiliations:** I Cirurgia de Cabeca e Pescoco, Instituto do Cancer do Estado de Sao Paulo (ICESP), Hospital das Clinicas HCFMUSP, Faculdade de Medicina, Universidade de Sao Paulo, Sao Paulo, SP, BR; II Cirurgia de Cabeca e Pescoco, Hospital das Clinicas HCFMUSP, Faculdade de Medicina, Universidade de Sao Paulo, SP, BR

This report aims to describe, for the first time in the literature, the use of transoral robotic surgery (TORS) associated with sentinel lymph node biopsy (SLNB), as well as to discuss the advantages and disadvantages of the two techniques when used separately or simultaneously. Technological developments in the medical field have led to amazing progress in surgery, and with the objective to perform a less invasive procedure with minimal complications and allow the early discharge of patients, the da Vinci System was introduced by Intuitive Surgical (Sunnyvale, CA, USA) in 1999. Since then, the number of robotic surgical devices has increased, demonstrating numerous advantages over conventional surgery, for example, fine precision and freedom of movement. TORS has emerged as the newest surgical alternative for the treatment of oropharyngeal squamous cell carcinoma (OPSCC) and has been growing steadily in popularity. TORS was first described in the literature in 2006 by Weinstein's team at the University of Pennsylvania for its successful use in animals, cadavers, and then in clinical practice, initially on oropharyngeal tumors and then on supraglottic tumors. In OPSCC, it is possible to use TORS for T1-2 tumors, but this kind of cancer can lead to precocious neck micrometastasis, which occurs in approximately 30% of patients with cN0 of the neck. Due to this probability, a prophylactic neck dissection is indicated; however, this is usually performed two weeks before the TORS procedure; therefore, the patient needs two admissions and two surgical procedures with anesthesia, and in 70% of the cases, no lymph node metastases are found. Moreover, there are some possible complications associated with the neck dissection, such as hematoma, spinal nerve palsy and lymphatic fistula. Because of these possibilities, three years ago, our group started to perform the SLNB of oral and oropharyngeal cancers with the same technique as that performed for melanoma or breast cancer. In patients with T1-2 N0 OPSCC, the TORS protocol along with simultaneous SLNB was introduced in order to minimize the side effects and the cost of this kind of surgery 1-3.

## METHODS

This study was approved from by the Ethics and Research Committee of the Faculty of Medicine of the Sao Paulo University (no. 438/13).

A consecutive, nonrandomized cohort of 40 patients were included in this study to evaluate the TORS method and whether SLNB could be performed. All patients had histopathologically diagnosed squamous cell carcinoma of the upper head and neck region and had neither regional metastases in cervical lymph nodes (cN0) nor distant metastases (cM0) as determined by clinical or radiological methods.

**Figure 1:**
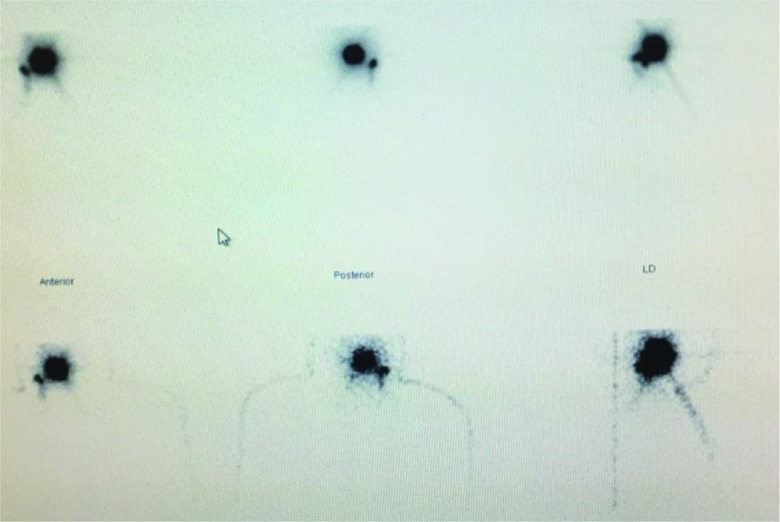
Dynamic scan sequence of 120-second frames for 30 min following the injection of 99Tec. Right tonsillar tumor with a sentinel lymph node at level II on the right side.

**Figure 2:**
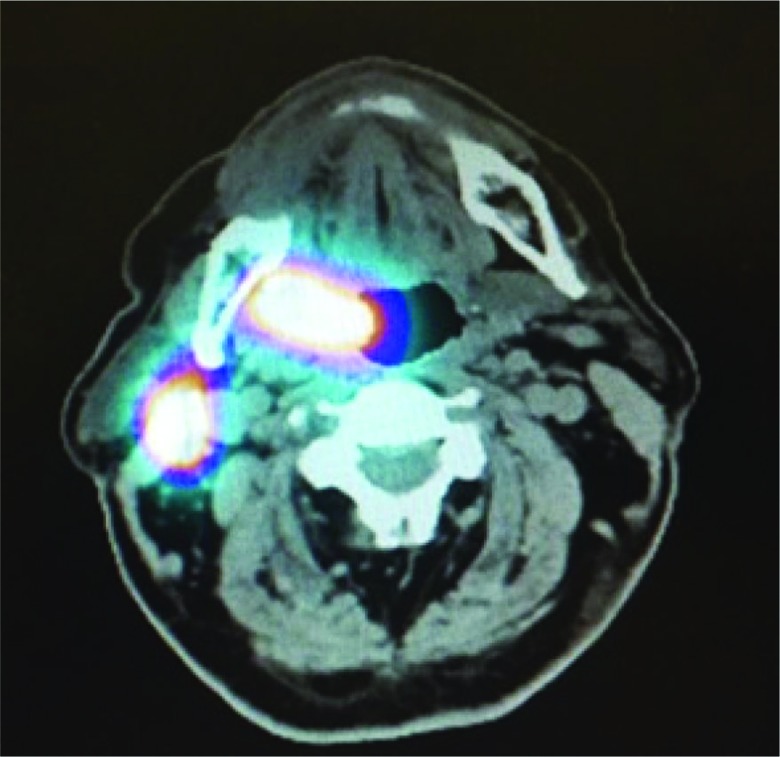
SPECT/CT and lymphoscintigraphy fusion. Right tonsillar tumor with a sentinel lymph node at level II on the right side.

The patients were placed under an orbiter camera, and topical anesthesia (10% lidocaine) was applied to their pharyngeal mucosa. Four peritumoral injections of 40 MBq technetium-99m (99Tec) nanocolloid dissolved in 0.2 ml of physiological saline solution were administered. The first dynamic scan sequence of 60 ten-second frames started immediately after the injection. This was followed by a second sequence of 120-second frames for 30 min. In addition, 5-minute scans were conducted from the anterior and lateral aspects on both sides after 30, 60 and 120 min (Figure 1). After the dynamic scan sequences were completed and a SPECT/ CT was performed (Figure 2), the sentinel lymph nodes (SLNs) were located using a gamma probe, and their positions were marked on the skin 3,4.

The day after SLN detection, we primarily performed the oropharyngeal tumor resection with TORS (a radical tonsillectomy). Then, we confirmed the location of the SLNs by using the gamma probe, which analyzed the uptake signal. After that, we made an incision and performed the lymph node resection. To confirm that the SLN was resected, the gamma probe signal on the bed of the surgical area should have diminished. Then, the sample was submitted for histopathological examination 3,4.

In accordance with international standards, the SLNs were then fixed in 10% formalin and embedded in paraffin. The tissues were then cut into sections of 150 mm and stained with hematoxylin and eosin 3,4.

### Case report

AASM is a 60-year-old female born in Boa Viagem, CE, Brazil and living in Diadema, SP and is a retired farmer. At the first consultation in July 2015, the patient had a one-year history of progressive dysphagia and sore throat. She had a smoking history of 50 pack-years, but quit one month before the appointment. She used to consume alcohol but stopped 6 months prior, and she has used a dental prosthesis for 40 years. At the physical exam, there was a reddened lesion involving the soft palate that was more prominent on the uvula and both anterior and posterior right pillars. There were no palpable cervical masses. Upon endoscopy, there was no involvement of the larynx or nasopharynx. Biopsy of the lesion on 07.16.2015 showed *in situ* squamous cell carcinoma. The face, neck and chest CT scan on 08.12.15 showed a thickening area and asymmetrical enhancement of the right soft palate, extending to the left tonsillar fossa and uvula. There were no suspicious cervical lymph nodes. The diagnosis was OPSCC, clinically staged as cT1 N0 M0.

On 10.02.2015, the patient underwent TORS, with the successful excision of a left tonsillar fossa tumor, with free frozen section margins. Simultaneous SLNB was performed with no complications. The final pathology report showed a moderately differentiated squamous cell carcinoma located on the left tonsillar fossa, with 1.2 cm in its greatest diameter and 0.3 cm thickness with free margins. No angiolymphatic or perineural invasion were found. Lymph node metastasis was identified in one of two SLNs removed with the left level II dissection. The final pathological staging was pT1 pNs1 M0. On 11.26.2015, the patient underwent a left modified radical neck dissection and a right elective selective neck dissection of levels I, II and III, because the lesion extended beyond midline. The pathological report found no additional metastatic lymph nodes. The patient had a great outcome after both surgeries without any complication. The hospital stay was short. and the follow-up period was uneventful.

## DISCUSSION

The first description of TORS was made in 2006 by Weinstein et al. at the University of Pennsylvania. Currently, TORS is widely used in head and neck surgery, especially for the surgical treatment of oropharyngeal cancers. Advantages include the avoidance of external incisions, improved functional outcomes, a reduced need for tracheostomy and gastrostomy, decreased blood loss, and shorter hospital stays 5,6. The main disadvantage of robotic surgery is the high financial cost, especially in developing countries 7.Robotic selective neck dissection via the postauricular facelift approach may be used for patients with clinically staged N0 tumors; however, this procedure should be strictly reserved for patients for whom cosmetic satisfaction is important because of its significantly increased operating time and cost 8,9. This technique is not routinely preformed in our environment.

To our knowledge, this is the first case reported in the literature in which combined TORS and open minimally invasive surgical technique (SLNB) was employed. The immediate follow-up period was uneventful; however, it is still impossible to evaluate the long term oncologic outcome of this patient. Nevertheless, the advantages of both minimally invasive techniques were achieved in the present case. SLNB is a reliable staging tool for T1/T2 cN0 OPSCC, without adverse effect on patient survival and with fewer complications. No late recurrences occurred in long-term follow-up. Close follow-up is mandatory for SN+ patients, who are at higher risk of nodal recurrence and have worse prognosis 3.

This is the first report of the combined, simultaneous use of two minimally invasive techniques, TORS and SLNB, in a patient with oropharyngeal cancer, with encouraging initial results.

## AUTHOR CONTRIBUTIONS

Kulcsar MA, Canovas NS, Araujo-Neto VJ, Kim JD performed the surgeries and wrote the manuscript. Cernea CR wrote the manuscript and approved the final version of the manuscript to be published.
